# Protective Effect of *Cyclotrichium niveum* on Gentamicin-Induced Nephrotoxicity in Rats

**DOI:** 10.3390/antiox15101322

**Published:** 2026-10-14

**Authors:** Gazihan Harun Özsayın, Özlem Yorulmaz-Özü, Umay Merve Güven, Serpil Demirci-Kayiran, Yurdun Kuyucu, Merve Nenni, Gülçin Dağlıoğlu, Olcay Kıroğlu, Fatma Peyman Ertuğ, Murat Türk, Burçin Ün, Eda Karabal-Kumcu, Mehmet Ata Seçilmiş

**Affiliations:** 1 Department of Pharmacology, Faculty of Medicine, Çukurova University, 01330 Adana, Turkey; 2 Department of Pharmaceutical Technology, Faculty of Pharmacy, Çukurova University, 01330 Adana, Turkey; 3 Department of Pharmaceutical Botany, Faculty of Pharmacy, Çukurova University, 01330 Adana, Turkey; 4 Department of Histology and Embryology, Faculty of Medicine, Çukurova University, 01330 Adana, Turkey; 5 Department of Analytical Chemistry, Faculty of Pharmacy, Çukurova University, 01330 Adana, Turkey; 6 Hospital Central Laboratory, Faculty of Medicine, Çukurova University, 01330 Adana, Turkey; 7 Ceyhan Vocational School of Higher Education, Çukurova University, 01920 Adana, Turkey; murturk@cu.edu.tr; 8 Pathology Laboratory Techniques, Health Services of Vocational School, Istanbul Kent University, 34406 Istanbul, Turkey; burcin.un@kent.edu.tr; 9 Institute of Addiction and Forensic Sciences, Çukurova University, 01330 Adana, Turkey

**Keywords:** nephrotoxicity, antioxidant treatment, kidney failure, *Cyclotrichium niveum*, gentamicin

## Abstract

Gentamicin is an aminoglycoside antibiotic and has therapeutic importance in the treatment of Gram-negative aerobic bacterial infections. However, its clinical use is limited by nephrotoxicity, in which oxidative stress plays an important role. In this study, we investigated the protective effects of *Cyclotrichium niveum*, an endemic plant with antioxidant properties, against gentamicin-induced nephrotoxicity in rats. Renal injury and protection were assessed using blood urea nitrogen (BUN) and serum creatinine as markers of renal function, malondialdehyde (MDA) as a marker of oxidative stress, and histopathological evaluation of renal cortical injury. We first characterized the chemical composition of *C. niveum* essential oil (EO) by GC-MS, identifying pulegone as its major constituent (79.6%). Next, we evaluated the in vitro antioxidant activity of EO and isolated pulegone using the DPPH assay. Their protective effects were then examined in vivo at two dose levels each (EO, 200 and 400 mg/kg/day; pulegone, 50 and 100 mg/kg/day, p.o.) in rats receiving gentamicin (100 mg/kg/day, i.m.) for 8 days. Both EO and pulegone exhibited DPPH radical-scavenging activity. Gentamicin induced pronounced renal injury, whereas co-administration of either EO or pulegone provided protection, with more pronounced effects at the higher doses. In particular, gentamicin increased BUN and creatinine 2.74- and 2.37-fold, respectively (*p* < 0.001 for both), and produced grade 4–5 cortical injury in all histologically evaluated animals. At the higher doses, EO and pulegone reduced BUN by 56% and 64%, respectively (*p* = 0.042 and *p* < 0.001), and MDA by 57% and 55%, respectively (*p* = 0.006 for both). Pulegone additionally reduced creatinine by 39% (*p* = 0.041). Both treatments significantly improved histopathological injury grade (*p* = 0.002 and *p* = 0.008 for EO and pulegone, respectively). Together, these findings demonstrate that *C. niveum* EO and pulegone attenuate gentamicin-induced renal injury across functional, oxidative, and structural measures, with the strongest protection observed at the higher doses. Their in vitro antioxidant activity and attenuation of lipid peroxidation in vivo support an antioxidant contribution to the nephroprotective response, while the activity of isolated pulegone identifies it as an important bioactive contributor to the protective effects of *C. niveum* EO.

## 1. Introduction

Gentamicin is an aminoglycoside antibiotic with established therapeutic value in the treatment of serious aerobic Gram-negative bacterial infections. However, its clinical utility is substantially limited by nephrotoxicity, which has been reported in approximately 10–20% of patients receiving the antibiotic [1,2,3,4]. Gentamicin accumulates preferentially in proximal tubular epithelial cells, where it disrupts lysosomal function, causes mitochondrial vacuolization, and initiates a cascade of cellular injury culminating in tubular necrosis, apoptosis, glomerular congestion, and impaired glomerular filtration [5,6,7,8,9]. As a result, gentamicin-induced renal injury is characterized by impaired renal function and prominent structural damage, particularly within the proximal tubules. The resulting acute kidney injury is often reversible after drug withdrawal, but recovery can be incomplete, especially after severe injury or in patients with pre-existing renal vulnerability [10]. Accordingly, appropriate dosing, renal-function monitoring, and therapeutic drug monitoring are important measures for reducing the risk of nephrotoxicity in susceptible patients. Strategies capable of protecting the kidney during gentamicin treatment are therefore of crucial importance, particularly when continued aminoglycoside therapy is clinically necessary.

Although the precise mechanisms underlying gentamicin-induced renal injury have not been completely elucidated, oxidative stress is recognized as a central mediator of its nephrotoxic effect. Gentamicin induces the generation of reactive oxygen species (ROS), including superoxide anion, hydrogen peroxide, and hydroxyl radicals, leading to lipid peroxidation and tubular cell injury, particularly in the proximal tubule [5,7,8,11,12,13,14,15]. Recent studies have further characterized the cellular pathways associated with this oxidative injury. Gentamicin-induced mitochondrial dysfunction is associated with several forms of regulated cell death. These include apoptosis in proximal tubular cells and necroptosis, which is prominent in the collecting duct [6]. More recently, ferroptosis, an iron-dependent form of cell death driven by lipid peroxidation, has also been implicated in gentamicin-induced renal injury, with reduced GPX4 and SLC7A11 and increased ACSL4 expression [16]. Gentamicin also suppresses the cytoprotective Nrf2/HO-1 axis [17] and activates NF-κB- and JNK1/iNOS-dependent inflammatory signaling, with increased expression of pro-inflammatory mediators including TNF-α, IL-1β, and iNOS [5,18,19]. Oxidative stress is therefore closely interconnected with mitochondrial dysfunction, inflammation, and apoptosis in gentamicin-induced renal injury. Accordingly, antioxidant and cytoprotective interventions targeting these processes represent potential strategies for limiting gentamicin-induced renal injury.

Consistent with this, gentamicin-induced nephrotoxicity has been attenuated by a range of antioxidant agents in experimental models, including vitamins, metal chelators, nitric oxide modulators, endogenous antioxidant enzymes, phenolic compounds, terpenoid-rich preparations, and other plant-derived bioactives [20,21,22,23,24,25,26,27,28,29,30,31,32,33,34,35,36,37,38,39,40]. A systematic meta-analysis of preclinical studies further demonstrated significant nephroprotection by antioxidants, with greater protective effects at higher doses and following oral rather than parenteral administration [41]. These findings support continued investigation of plant-derived antioxidants as potential nephroprotective and/or anticancer agents, given their chemical diversity, reported biological activities, and long history of traditional use [42,43,44,45,46].

*Cyclotrichium niveum*, a perennial aromatic species of the Lamiaceae family, is endemic to eastern Anatolia, Turkey, where it is known locally as “dağ nanesi” [47,48,49]. The plant has traditionally been used in the preparation of soup and herbal tea and as a food flavouring [50]. Beyond its ethnobotanical relevance, *C. niveum* has attracted scientific interest because of its reported biological activities. Previous studies have shown that *C. niveum* possesses antispasmodic effects on rabbit bladder and rat ileum smooth muscle [51], while its polyphenolic constituents have demonstrated total antioxidant, hydrogen peroxide-scavenging, superoxide dismutase-like [52], antimicrobial [53], and anticancer activities in vitro [54,55]. These antioxidant properties suggest that *C. niveum* may possess cytoprotective potential under conditions of oxidative injury. However, its nephroprotective potential has not previously been evaluated in vivo. Given the contribution of oxidative stress to gentamicin nephrotoxicity, the reported antioxidant activity of *C. niveum* provides a strong motivation to investigate this endemic species in the present model.

The essential oil of *C. niveum* is characterized by a high relative abundance of pulegone, a monocyclic monoterpene ketone [47]. Previous studies have reported various biological activities of essential oil constituents, including antioxidant effects [56], and pulegone itself has also been associated with such activities [57]. Given its predominance in the essential oil composition and its reported biological activities, pulegone represents a logical candidate as a major bioactive contributor to the potential nephroprotective activity of *C. niveum*. Evaluating pulegone independently alongside the essential oil may therefore help determine whether a major defined constituent contributes to the renal cytoprotective activity of the essential oil and advance the mechanistic understanding of *C. niveum*’s pharmacological profile.

We designed this study to investigate the possible protective effects of *C. niveum* essential oil and isolated pulegone against gentamicin-induced nephrotoxicity in rats. By assessing renal function, oxidative stress, and histopathological injury, we aimed to determine whether the essential oil and pulegone attenuate gentamicin-induced renal damage and to evaluate the contribution of pulegone to the protective activity of *C. niveum* essential oil.

## 2. Materials and Methods

**Collection of the Plant Specimens.** *Cyclotrichium niveum* (Boiss.) Manden. & Scheng. was collected in Adıyaman Nemrut Mountain, Pınarbaşı village of Sincik district, 1083 m a.s.l., in July 2017. The species was identified by Prof. Dr. Ahmet Zafer Tel, and a voucher specimen was deposited in the Herbarium of Adıyaman University (Herbarium number 1382).

**Preparation of Essential Oils.** Twenty-five grams of air-dried leaves were subjected to hydrodistillation for 3 h using a Clevenger-type apparatus to obtain the essential oil (EO). Oil yield was calculated relative to dry leaf mass as Y (%) = (grams of extract/grams of dried material) × 100. The yield of EO, determined in quadruplicate, was 4.07 ± 0.36%. The EO was emulsified in soybean oil for oral administration. Doses of 200 mg/kg and 400 mg/kg were administered to experimental animals by oral gavage using an orogastric catheter.

**Formulations of Pulegone.** Pulegone standard (CAS 89-82-7, molar mass 152.23 g/mol) was obtained from Merck KGaA (Darmstadt, Germany) and emulsified in soybean oil. Doses of 50 mg/kg and 100 mg/kg were administered to experimental animals by oral gavage.

**Dose selection.** The low doses of both the essential oil (EO) and pulegone were selected from preliminary dose-finding experiments as the lowest doses at which nephroprotective activity began to appear, and the high doses were set at twice the corresponding low doses to examine dose–response behavior.

**GC-MS Analysis.** Chemical composition of the essential oil was determined using a Finnigan-Trace GC-MS equipped with an autosampler. One microliter of sample was injected in split mode (split ratio 1:50) onto a Thermo TR-MS-5 capillary column (5% phenyl-95% dimethylpolysiloxane, 0.25 mm i.d. × 60 m, film thickness 0.25 µm) (Thermo Fisher Scientific, Waltham, MA, USA). Helium was used as the carrier gas at a flow rate of 1.0 mL/min. The oven temperature was programmed from 50 °C to 160 °C at 3 °C/min, held for 3 min, then increased at 5 °C/min to 250 °C and held for 10 min. The injection port temperature was 250 °C, and the ionization voltage was 70 eV, with a mass range of *m*/*z* 41–400 a.m.u. Components were tentatively identified by matching mass spectra against the National Institute of Standards and Technology (NIST) spectral library, and relative abundance was determined by peak area integration.

**Antioxidant Activity.** Antioxidant capacity of the EO and pulegone preparations was assessed using the 2,2-diphenyl-1-picrylhydrazyl (DPPH) radical scavenging assay, adapted to a 96-well plate format [58]. The assay is based on the reduction of DPPH (purple-violet) to its yellow-colored reduced form upon reaction with hydrogen-donating antioxidants, monitored at 517 nm. Forty microliters of 0.1 mM DPPH solution (in ethanol) were added to 120 µL of test substance prepared at varying concentrations in ethanol from Merck KGaA (Darmstadt, Germany). Ascorbic acid (from Sigma-Aldrich, St. Louis, Missouri, USA) served as the positive control [59]. Plates were incubated at 25 °C in the dark for 30 min, and absorbance was read at 517 nm against an ethanol blank using a microplate reader (Thermo Scientific™ Multiskan™ Sky Microplate Spectrophotometer, Waltham, MA, USA). Each assay was performed in triplicate. Percent DPPH scavenging activity was calculated as:

% DPPH Scav. Act. = [(A_Control_ − A_Sample_)/A_Control_] × 100,

where A_Control_ is the absorbance of the vehicle, and A_Sample_ is the absorbance of the test sample. IC50 values were determined from plots of percent DPPH scavenging activity against concentration.

**Experimental Protocol.** Male Wistar Albino rats (200–300 g) were obtained from the Health Sciences Experimental Application and Research Center (SABIDAM). Animals were housed in polycarbonate cages at 24 ± 2 °C under a 12 h light/12 h dark cycle. The study was approved by the Animal Experiments Local Ethics Committee of Çukurova University Health Sciences Experimental Application and Research Center (SABIDAM; ethical protocol code 3–13; approval date 4 July 2018).

Eight rats were initially assigned to each experimental group. Some animals were lost during the course of the experiment, leaving 6–8 animals per group at the end of the study. All treatments were administered for eight days: (i) Control group (*n* = 6), receiving normal diet and drinking water; (ii) Gentamicin group (*n* = 6), receiving gentamicin at 100 mg/kg/day, intramuscularly (im); (iii) Gentamicin + EO-L group (*n* = 7), receiving gentamicin (100 mg/kg/day, im) co-administered with EO at 200 mg/kg/day, orally (gavage); (iv) Gentamicin + EO-H group (*n* = 8), receiving gentamicin (100 mg/kg/day, im) co-administered with EO at 400 mg/kg/day, orally (gavage); (v) Gentamicin + Pul-L group (*n* = 6), receiving gentamicin (100 mg/kg/day, im) co-administered with pulegone at 50 mg/kg/day, orally (gavage); and (vi) Gentamicin + Pul-H 100 group (*n* = 8), receiving gentamicin (100 mg/kg/day, im) co-administered with pulegone at 100 mg/kg/day, orally (gavage).

24 h after the final administration on day 8, animals were anesthetized with ketamine (Ketalar^®^, 40 mg/kg) (Pfizer, Lüleburgaz/Kırklareli, Türkiye) and xylazine hydrochloride (Basilazin 2%, 5 mg/kg) (Bavet, İstanbul, Türkiye) and sacrificed. Intracardiac blood was collected for biochemical analysis, and the left kidney was removed via a midline abdominal incision for histopathological examination.

**Biochemical Analysis.** Blood for biochemical analysis was collected by direct cardiac puncture into 3.5 mL serum-separator (gel) Vacutainer tubes (Becton Dickinson (BD), Franklin Lakes, NJ, USA). Samples were centrifuged at 11,000 rpm for 10 min, and serum was separated and stored at −20 °C until analysis. Serum creatinine and blood urea nitrogen (BUN) were measured in the Çukurova University Biochemistry Central Laboratory on a Beckman Coulter AU5800 automated clinical chemistry analyzer (Beckman Coulter Inc., Brea, CA, USA), following the manufacturer’s instructions and routine operating and quality-control procedures. Creatinine was determined by the kinetic Jaffé colorimetric method, and BUN by an enzymatic kinetic UV urease-glutamate dehydrogenase (urease-GLDH) method. Serum malondialdehyde (MDA) was quantified using a rat-specific MDA enzyme-linked immunosorbent assay kit (SunRed Biological Technology Co., Ltd., Shanghai, China) according to the manufacturer’s instructions. The effective sample size for each biochemical marker reflects the number of measurements delivered by the laboratory after routine quality-control procedures and therefore may differ from the number of animals initially assigned to some groups (6–8 measurements for BUN and creatinine and 4–6 measurements for MDA before statistical outlier handling).

**Histopathological Examination.** Renal tissue samples were fixed in 10% neutral formalin for 3 days and processed using a Leica TP 1020 automated tissue processor (Leica Biosystems, Nussloch, Germany), followed by paraffin embedding after successive dehydration steps. Sections of 5 µm thickness were stained with hematoxylin and eosin (H&E) and examined and photographed using an Olympus BX50 light microscope (Olympus Optical Co., Ltd., Tokyo, Japan). From each group, five animals were randomly selected for histopathological assessment. Sections were scored by an observer blinded to the treatment group, evaluating glomerular necrosis, tubular degeneration and necrosis, tubular dilatation, intratubular cast formation, and interstitial inflammation. Injury severity was graded on a six-tier scale: grade 0, normal; grade 1, minimal focal changes; grade 2, histopathological changes in less than 25% of the cortex; grade 3, 25–50%; grade 4, 50–75%; grade 5, more than 75% [60].

**Statistical Analysis.** Prior to statistical testing, available values for each biochemical marker (BUN, creatinine, MDA) were screened for outliers within each treatment group using the interquartile range (IQR) method, with values falling below Q1 − 1.5 × IQR or above Q3 + 1.5 × IQR flagged and excluded from subsequent statistical analysis; flagged values were retained in graphical representations but excluded from group-level comparisons. The decision to exclude outliers was made to avoid potential cases where, in modest sample sizes like ours, a single aberrant observation may retain substantial leverage on group rank sums. To assess the consequences of this exclusion procedure, we performed a sensitivity analysis in which the flagged values were retained. We report the exact excluded outliers in Supplementary Note S1. Final group sizes after any missing measurements and the exclusion of flagged values were 6–8 for BUN, 4–7 for creatinine, and 3–6 for MDA per treatment group.

Because of the small and unequal group sizes, biochemical results were analyzed using nonparametric tests. For each biochemical marker, overall group differences were assessed using the Kruskal–Wallis test, followed by Dunn pairwise comparisons. Comparisons were divided into four biological hypothesis families: disease induction (Control vs. Gentamicin), treatment efficacy (each treatment vs. Gentamicin), comparison with the healthy control (each treatment vs. Control), and dose–response (low vs. high dose within each preparation). Dunn *p*-values were adjusted using the Holm test within each biochemical marker and hypothesis family. Descriptive statistics are reported as medians with interquartile ranges (Q1–Q3), and fold changes were calculated as the ratio of the treatment-group median to the corresponding reference-group median. Pairwise effect sizes were expressed as δ (Cliff’s delta), with confidence intervals where estimable, and Kruskal–Wallis effect sizes were summarized using ε^2^ (epsilon squared). We repeated the same rank-based comparisons for a sensitivity analysis, this time also including the previously excluded statistical outliers, to assess the robustness of the primary findings to the statistical outlier handling. As a supportive robustness analysis, the same biochemical pairs were also evaluated using Welch two-sample comparisons.

Histopathological grade distributions were compared across the six main groups using the Kruskal–Wallis test, followed by Dunn comparisons for disease induction, treatment efficacy versus Gentamicin, comparison with Control, and low-versus-high dose contrasts. Dunn *p*-values were Holm-adjusted for multiple testing within each biological comparison family.

For all statistical tests, a *p*-value (or Holm-adjusted *p*-value, where applicable) of less than 0.05 was considered statistically significant. Statistical analyses were performed in R (v4.6.0) using the rstatix and effsize packages in addition to base packages.

**Data visualization.** Figures were generated in R (v4.6.0) using the ggplot2, ggpubr, and related packages, and exported in PDF, PNG, and SVG formats, which were then combined using Adobe Illustrator v30.8.2.

## 3. Results

In this study, we investigated whether *C. niveum* preparations could confer renal protection in a gentamicin-induced nephrotoxicity model in rats. To this end, we designed a study spanning in vitro chemical characterization and antioxidant screening through to in vivo functional and histopathological assessment.

### 3.1. Chemical Composition and Antioxidant Activity of C. niveum Essential Oil

**Figure 1:**
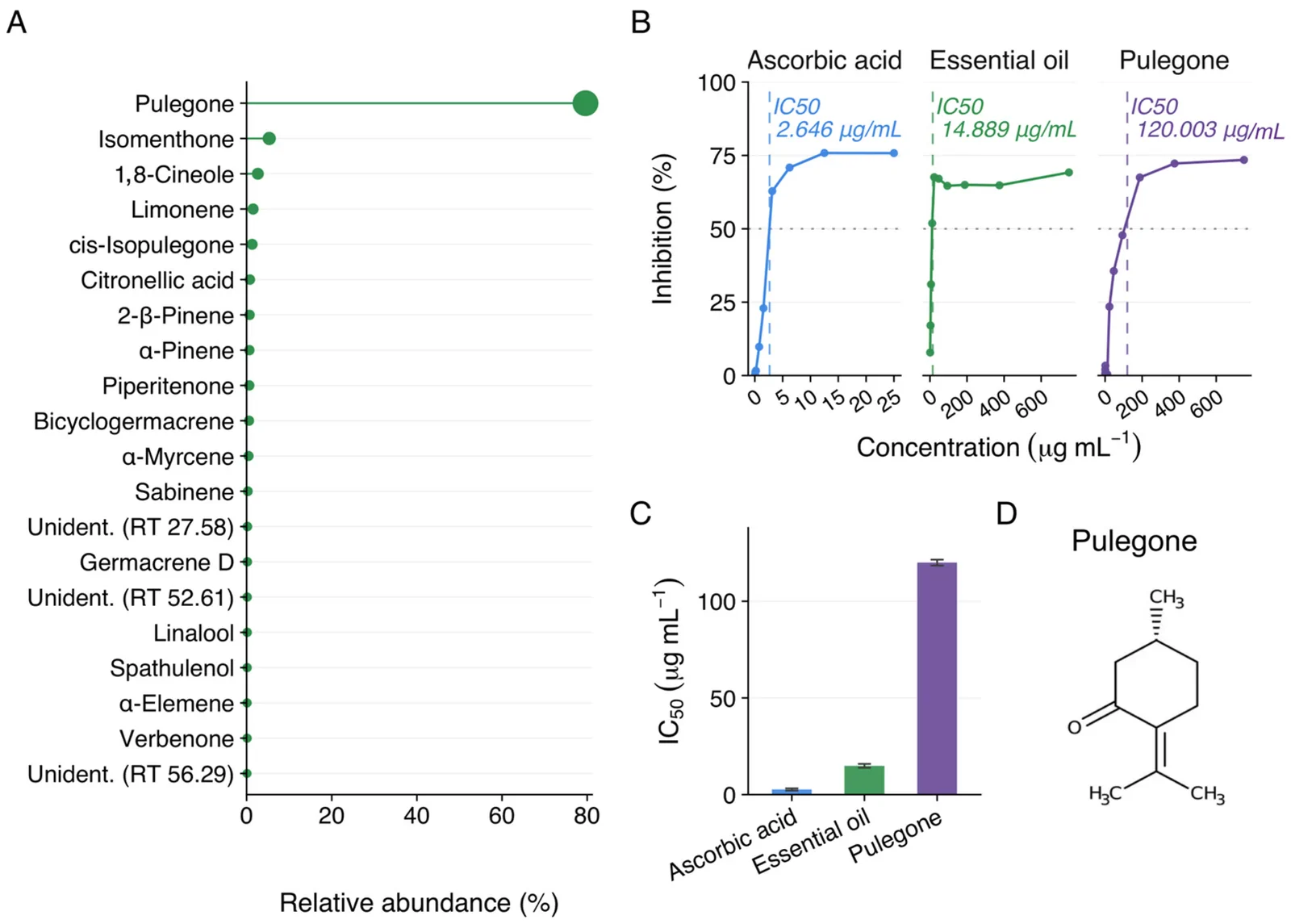
Chemical composition and antioxidant activity of *C. niveum* essential oil and pulegone. (**A**) Relative abundance (%) of the 20 detected components of the essential oil (EO), ranked by abundance; pulegone constitutes the dominant component (79.6%). (**B**) DPPH radical scavenging activity (% inhibition) as a function of concentration (µg/mL) for ascorbic acid (positive control), EO, and pulegone. Dashed vertical lines and labels indicate the IC50 for each preparation; the dotted horizontal line marks 50% inhibition. (**C**) Summary IC50 values (µg/mL) for each preparation. Bars represent mean ± SD; lower IC50 indicates greater antioxidant potency. (**D**) Structure of pulegone.

The essential oil of *C. niveum* was obtained by hydrodistillation of dried aerial parts, yielding a mean of 4.07% (*v*/*w*), and its chemical composition was determined by GC-MS analysis. A total of 20 compounds were detected, accounting for 99.62% of the total oil composition (Figure 1A). Pulegone was overwhelmingly the dominant constituent, comprising 79.6% of the total oil. The remaining identified compounds were present at considerably lower abundances, with isomenthone (5.32%), 1,8-cineole (2.64%), limonene (1.52%), and citronellic acid (0.77%) constituting the most notable minor constituents. Three compounds could not be identified by library matching.

The antioxidant capacity of the EO and isolated pulegone was assessed by the DPPH free radical scavenging assay [58], with ascorbic acid as the positive control. Both preparations exhibited concentration-dependent radical scavenging activity across the tested concentration ranges (Figure 1B). The EO showed a stronger antioxidant activity, reaching 50% DPPH inhibition at an IC50 of 14.89 ± 0.99 µg/mL, than pulegone (IC50 = 120.00 ± 1.52 µg/mL; Figure 1C). Both IC50 values were higher than that of the ascorbic acid positive control (2.65 ± 0.57 µg/mL), by approximately 5.6-fold for EO and 45.4-fold for pulegone. Notably, the EO showed substantially greater radical scavenging potency than isolated pulegone despite pulegone comprising 79.6% of its composition, suggesting that minor constituents may make a disproportionate contribution to the in vitro antioxidant profile of the oil [61].

### 3.2. Essential Oil and Pulegone Attenuate Gentamicin-Induced Renal Injury

To evaluate nephroprotective potential in vivo, gentamicin was administered at 100 mg/kg/day for 8 consecutive days to induce nephrotoxicity, a well-established model of aminoglycoside-induced renal injury [15,62]. EO was co-administered with gentamicin at 200 mg/kg/day (low dose, EO-L) and 400 mg/kg/day (high dose, EO-H), and pulegone was co-administered with gentamicin at 50 mg/kg/day (low dose, Pul-L) and 100 mg/kg/day (high dose, Pul-H). Renal function was then assessed by measuring blood urea nitrogen (BUN) and serum creatinine [63], and malondialdehyde (MDA) was measured as an indicator of lipid peroxidation and oxidative stress [9,28,36,64].

**Figure 2:**
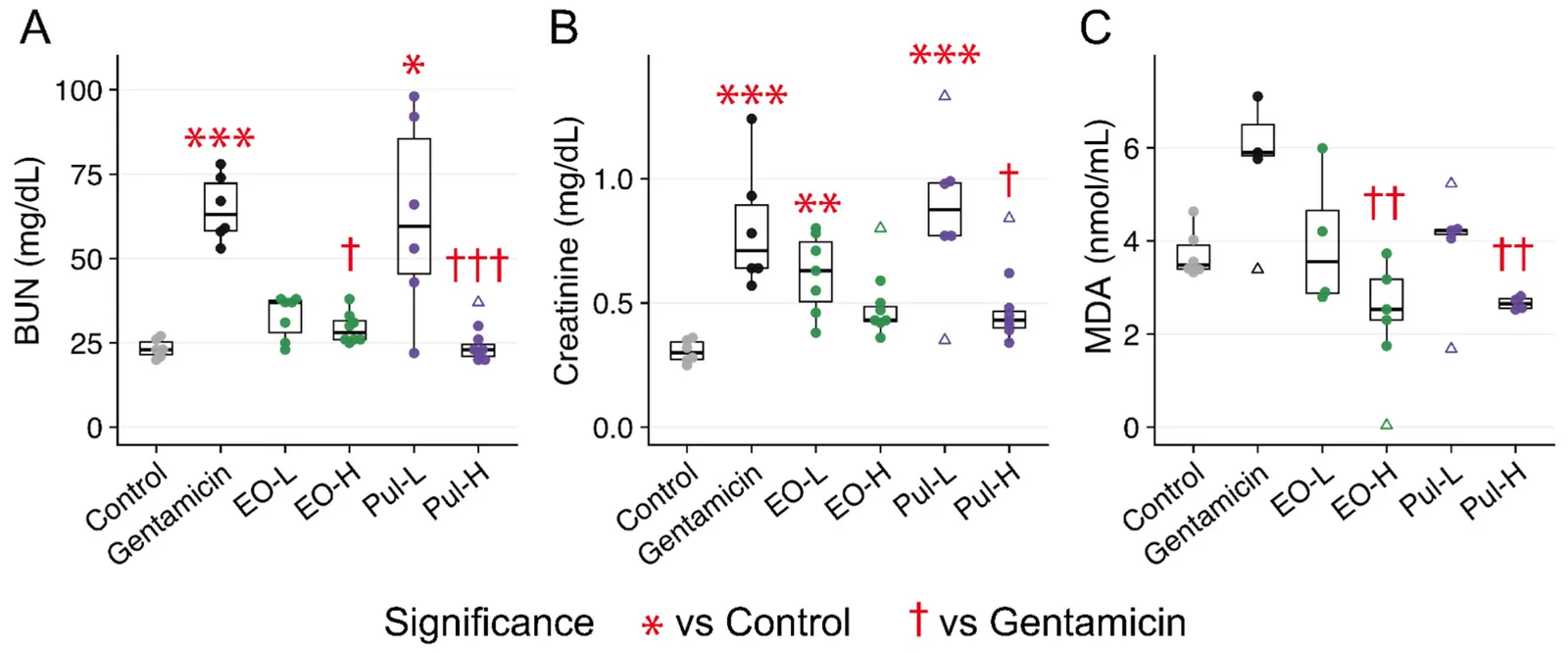
Effects of *C. niveum* essential oil and pulegone on biochemical markers of gentamicin-induced nephrotoxicity. (**A**) Blood urea nitrogen (BUN, mg/dL), (**B**) serum creatinine (mg/dL), and (**C**) malondialdehyde (MDA, nmol/mL) across color-coded Control (gray), Gentamicin (black), and treatment groups (EO-L/EO-H, green; Pul-L/Pul-H, purple). Filled circles represent individual observations, whereas open triangles indicate values flagged by the IQR criterion and excluded from the primary analysis. Boxes show the median and interquartile range. Red asterisks indicate significant comparisons with Control and red daggers indicate significant comparisons with Gentamicin; only statistically significant comparisons are displayed (* or †, *p* < 0.05; ** or ††, *p* < 0.01; *** or †††, *p* < 0.001).

Gentamicin administration produced a significant deterioration in renal function, with median BUN increasing from 23 mg/dL in the Control group to 63 mg/dL in the Gentamicin group, corresponding to a 2.74-fold (174%) increase (*p* < 0.001 ***, Figure 2A). Similarly, median creatinine increased from 0.30 to 0.71 mg/dL, representing a 2.37-fold (137%) increase (*p* < 0.001 ***, Figure 2B). Gentamicin also increased median MDA from 3.48 to 5.90 nmol/mL, an approximately 1.69-fold (69%) increase in oxidative stress, although this difference did not reach statistical significance (*p* = 0.090, Figure 2C).

Co-treatment with EO ameliorated the biochemical alterations induced by gentamicin, with the improvements generally becoming more pronounced at the higher dose (Figure 2). At the lower dose, EO reduced median BUN from 63 to 37 mg/dL (Figure 2A), and MDA from 5.90 to 3.55 nmol/mL (Figure 2C), corresponding to reductions of approximately 41% and 40%, respectively. Creatinine was also lower by approximately 11%, decreasing from 0.71 to 0.63 mg/dL (Figure 2B). Although these changes did not reach statistical significance compared with the Gentamicin group, BUN and MDA were numerically lower than with gentamicin treatment alone. Creatinine, however, remained significantly elevated relative to Control at EO-L (*p* = 0.003 **, Figure 2B), indicating that renal functional recovery was incomplete at this dose. At the higher dose (EO-H), the protective effects were more pronounced. BUN decreased by approximately 56%, from 63 to 28 mg/dL (*p* = 0.042 †, Figure 2A), and MDA decreased by approximately 57%, from 5.90 to 2.53 nmol/mL (*p* = 0.006 ††, Figure 2C). Creatinine was similarly reduced by approximately 39%, from 0.71 to 0.43 mg/dL (Figure 2B), although this comparison remained just above the statistical significance threshold (*p* = 0.058). Thus, the higher EO dose produced substantial improvement across renal function and lipid peroxidation. BUN, creatinine, and MDA were also numerically lower at EO-H than EO-L, although these direct dose comparisons did not reach statistical significance (Figure S1 and Supplementary Table S1).

Pulegone showed a clear dose-related difference in its protective effects against gentamicin-induced renal injury. At the low dose, median BUN was approximately 6% lower than in the Gentamicin group (59.5 vs. 63 mg/dL; *p* = 0.480, Figure 2A), while creatinine was 23% higher (0.875 vs. 0.710 mg/dL; *p* = 0.676, Figure 2B). Both BUN and creatinine remained significantly elevated relative to Control (*p* = 0.010 * and *p* < 0.001 ***, respectively). In contrast, MDA decreased by approximately 28%, from 5.90 to 4.22 nmol/mL compared to the Gentamicin group (Figure 2C), although this difference did not reach statistical significance (*p* = 0.437). Thus, the lower pulegone dose produced a reduction in lipid peroxidation without statistical evidence of improvement in renal functional markers. At the high dose, pulegone (Pul-H, 100 mg/kg) significantly reduced BUN by 63.5% compared with the Gentamicin group (*p* = 0.00028 †††) and reduced MDA by 55.1% (*p* = 0.0060 ††). Creatinine was reduced by 39.4% compared to the Gentamicin group (*p* = 0.041 †). Direct comparison of the two pulegone doses showed significantly lower BUN (*p* = 0.0024), creatinine (*p* = 0.0136), and MDA (*p* = 0.0383) with Pul-H, demonstrating a pronounced dose-related difference.

Given the relatively small group sizes inherent to animal experiments, we further examined the robustness of the biochemical findings using complementary statistical approaches. Sensitivity analyses retaining the statistical outliers and supportive Welch analyses were therefore considered alongside the primary nonparametric analysis (Supplementary Note S1 for the statistical outliers excluded from the primary analyses; Supplementary Tables S2 and S3 for the sensitivity and Welch results, respectively). These analyses generally supported the principal treatment patterns, although some individual comparisons, particularly those involving creatinine and the pulegone dose contrasts, were sensitive to the analytical approach.

### 3.3. Histopathological Analysis Confirms Structural Renal Protection

**Figure 3:**
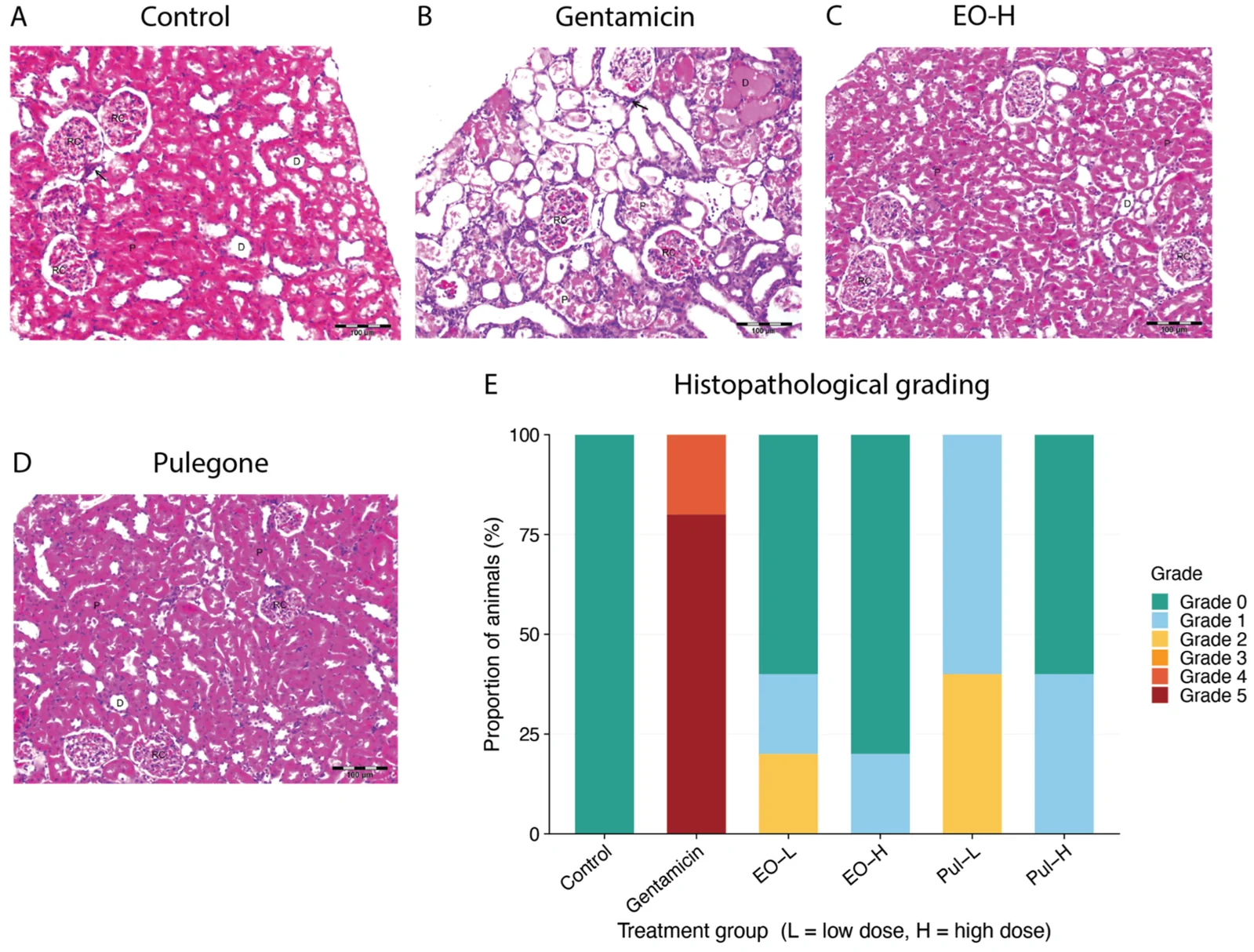
Histopathological evaluation of renal injury. Representative photomicrographs (H&E staining, Bars = 100 µm) of renal cortex and medulla from the (**A**) Control, (**B**) Gentamicin, (**C**) EO-H, and (**D**) Pul-H groups, respectively. On these photomicrographs, D denotes distal tubule, P refers to proximal tubule, and RC marks renal corpuscle. Black arrows point to macula densa and leukocyte infiltration in the Control and Gentamicin panels, respectively. (**E**) Distribution of histopathological injury grades (0–5; see Section 2) across the treatment groups, expressed as the proportion of animals per group (*n* = 5 per group).

Histopathological examination of renal tissue was performed to assess renal dysfunction and protection across all groups, using a six-tier grading system [60]: grade 0 (normal), grade 1 (minimal focal changes), grade 2 (histopathological changes in less than 25% of cortex), grade 3 (25–50%), grade 4 (50–75%), and grade 5 (more than 75%). From each group, five animals were randomly selected for histopathological assessment. Representative micrographs for the control, gentamicin, EO-H, and Pul-H groups are shown in Figure 3A–D, respectively, and the distribution of grades across the main experimental groups is presented in Figure 3E.

Renal cortex and medulla appeared entirely normal in control animals, with all five animals receiving grade 0 (Figure 3A,E). Gentamicin administration produced severe structural injury: renal corpuscles were generally preserved, but proximal tubular epithelial cells showed pronounced desquamation from the basal lamina, swelling, and lysis, with eosinophilic cellular remnants in the luminal space. Hyaline casts were observed in distal tubular lumina, and congestion with leukocyte infiltration was evident throughout the cortex and medulla. Four of five gentamicin-treated animals received grade 5 and one received grade 4, reflecting diffuse injury exceeding 50% of the cortex in all animals (Figure 3B,E). The overall grade distribution differed significantly across groups (*p* = 0.00069).

EO-L and EO-H both produced significant reductions in histopathological grade versus the gentamicin group (*p* = 0.0094 and *p* = 0.0023, respectively). In the EO-H group, four of five animals received grade 0 and one received grade 1 (Figure 3E), with renal architecture appearing similar to controls (Figure 3C). EO-L showed a broader distribution with three animals at grade 0, one at grade 1, and one at grade 2 (Figure 3E), with a more favorable grade distribution at the higher dose. However, the direct EO-L versus EO-H comparison did not reach statistical significance (*p* = 0.535; Supplementary Table S4).

Pul-H produced a significant reduction in grade distribution versus the gentamicin group (*p* = 0.0075), with three of five animals at grade 0 and two at grade 1 (Figure 3D,E), exhibiting a structural profile closely resembling the control and consistent with the marked reductions in BUN and MDA observed in the biochemical analysis (Figure 2). Pul-L did not reach significance versus the gentamicin group, with animals distributed across grades 1 and 2 (Figure 3E), in line with the absence of significant functional protection at this dose. Although the grade distribution had a healthier appearance at the higher dose, the direct Pul-L versus Pul-H comparison did not reach statistical significance (*p* = 0.192; Supplementary Table S4).

Taken together, the histopathological findings support the biochemical results: EO-H and Pul-H produced the most consistent structural and functional renal protection, while EO-L also showed substantial preservation despite more modest biochemical effects, and pulegone at the low dose provided partial protection.

In addition to the chemically characterized EO and isolated pulegone, we evaluated an ethanol extract (EE) to determine whether antioxidant and nephroprotective activity was also evident in another *C. niveum* preparation. EE showed concentration-dependent DPPH radical-scavenging activity and attenuated several biochemical changes induced by gentamicin, with significant reductions in BUN at both doses and in MDA at the higher dose. Histopathological examination also showed a notable shift from the severe grade 4–5 injury observed with the Gentamicin group to grade 1–2 injury in all EE-treated animals. The complete biochemical and histopathological findings, together with the corresponding statistical analyses, are presented in Supplementary Note S2 and Figure S2.

Across the in vivo experiments, EO and pulegone attenuated gentamicin-induced renal injury, with the most consistent biochemical and structural protection observed at the higher doses. EO also showed substantial histopathological protection at the lower dose despite more modest biochemical effects, while the protective effects of pulegone were considerably more pronounced at the higher dose in both biochemical and histopathological assessment. Together with the supplementary findings obtained with EE, these results demonstrate protective activity across different *C. niveum* preparations, and identify EO and its major constituent pulegone as a principal focus for further mechanistic interpretation.

## 4. Discussion

In this study, we for the first time show the protective effect of *C. niveum* on gentamicin-induced nephrotoxicity in vivo in rats, and identify pulegone as a major candidate contributor to the observed protection.

Gentamicin is among the most widely used aminoglycoside antibiotics in clinical practice, yet its nephrotoxic potential significantly limits its therapeutic use. Its preferential accumulation in proximal tubular epithelial cells causes a multifactorial injury involving mitochondrial dysfunction, oxidative stress, inflammation, and cell death [1,2,3]. The resulting nephrotoxicity is manifested functionally by elevations in BUN and creatinine, biochemically by increased lipid peroxidation reflected by MDA, and structurally by proximal tubular damage and progressive cortical involvement [4,5,6]. In our study, the findings across these three aspects closely recapitulated the established patterns of gentamicin-induced renal injury.

When co-administered with gentamicin, both the *C. niveum* essential oil and isolated pulegone attenuated the resulting renal injury. The observed decreases in BUN and creatinine indicate improvements in the functional damage induced by gentamicin, while the accompanying reductions in MDA indicate attenuation of lipid peroxidation associated with the injury. Renal morphology was likewise preserved, with substantially less tubular damage than that observed with gentamicin treatment alone, collectively confirming the plant’s nephroprotective potential. The strongest findings were obtained at the high doses of *C. niveum* essential oil and pulegone, where the protective effects were most pronounced and supported by the clearest statistical evidence, whereas the lower doses produced more limited responses. This dose-related pattern is consistent with the previous literature [41], which identified dose as an important factor influencing the nephroprotective effects of antioxidant interventions in experimental gentamicin nephrotoxicity. In the present study, this pattern was particularly evident for pulegone, whereas the distinction between the two essential oil doses was less pronounced.

The attenuation of lipid peroxidation provides an important mechanistic connection between the nephroprotective effects observed in this study and the established pathophysiology of gentamicin-induced renal injury. Oxidative stress is a major component of gentamicin-induced tubular damage [2,3,5], and both the *C. niveum* essential oil and pulegone showed radical-scavenging activity in vitro. Notably, the significant decreases in MDA at the high doses of both the essential oil and pulegone support attenuation of lipid peroxidation as part of their nephroprotective effects. This is consistent with previous experimental evidence showing that preventing oxidative stress can attenuate gentamicin nephrotoxicity [5,9,26,28,30,36].

The relationship between in vitro antioxidant capacity and in vivo antioxidant-mediated protection, however, is not strictly linear. In our study, the essential oil was approximately 8-fold more potent than pulegone in the DPPH assay, whereas the high doses of the essential oil and pulegone produced similar reductions in MDA levels. This difference can be explained by the distinct pharmacological conditions governing antioxidant activity in vivo: DPPH measures antioxidant activity in a cell-free chemical system and does not account for the pharmacokinetic and pharmacodynamic factors that determine antioxidant activity in vivo, including absorption, bioavailability, metabolism, renal distribution, and engagement of endogenous antioxidant pathways. Moreover, isolated pulegone and pulegone administered as a constituent of the essential oil are not pharmacologically equivalent, since the other essential oil constituents may influence its biological activity and may themselves contribute to the antioxidant response. The stronger DPPH activity of the essential oil may therefore reflect contributions from these minor constituents and their interactions, while the comparable MDA responses of the high-dose essential oil and pulegone show that this difference in in vitro potency does not translate proportionally to antioxidant-mediated effects in renal tissue. The present study cannot resolve whether the interactions among essential oil constituents are additive, synergistic, or antagonistic, which would require constituent-resolved and combination studies.

At the same time, MDA reflects lipid peroxidation rather than the broader renal antioxidant response. Establishing how the essential oil and pulegone modulate this antioxidant response would require a further assessment of endogenous antioxidant systems, including SOD, CAT, GPx, and GSH, together with redox-regulatory pathways such as Nrf2/HO-1 and early indicators of acute kidney damage such as KIM1 and NGAL [5,13,15,17,38,62]. Such measurements would help distinguish direct radical-scavenging effects from modulation of endogenous antioxidant mechanisms and more precisely define how attenuation of oxidative stress contributes to the observed nephroprotection in the present model.

Throughout the study, we followed a cautious statistical approach to account for the modest group sizes and the influence that individual observations can exert on statistical inference in animal experiments. We therefore used nonparametric analyses as the primary approach and presented these findings alongside supplementary statistical analyses, enabling assessment of the consistency of the observed effects across different statistical approaches. Importantly, renal function assessed by BUN and creatinine, lipid peroxidation reflected by MDA, and histopathological injury represent distinct but complementary aspects of gentamicin nephrotoxicity, and their overall patterns were consistent with the conclusions presented in this study. The agreement across these complementary endpoints therefore strengthens the overall biological interpretation of the treatment effects, while the statistical and experimental limitations should be considered when evaluating individual comparisons.

The present study has limitations in the mechanistic resolution of the observed nephroprotective effects. The in vitro antioxidant assessment was limited to the DPPH assay, and complementary methods such as ABTS, FRAP, and ORAC, together with cell-based ROS assays, would provide a broader assessment of antioxidant activity [65]. Although the present results demonstrate functional, biochemical, and structural protection against gentamicin-induced renal injury, the cellular and molecular pathways underlying these effects were not directly assessed. In particular, further investigation of inflammatory and cell-death pathways implicated in gentamicin nephrotoxicity, as well as mitochondrial function, would help define the mechanisms involved. Moreover, in addition to the commonly used clinical markers of renal function such as BUN and creatinine, more sensitive biomarkers of renal injury such as KIM-1 and NGAL, together with urinary protein excretion and urine volume, would complement the present assessment [7,25,63]. Assessment of inflammatory mediators and signaling pathways, including NF-κB, TNF-α, and IL-1β, would further strengthen the mechanistic characterization. Finally, pharmacokinetic measurements were not performed, and the systemic and renal exposure to pulegone, either administered alone or as a constituent of the essential oil, therefore remains to be studied.

This study establishes that *C. niveum* preparations attenuate gentamicin-induced nephrotoxicity with an important contribution from antioxidant activity to their protective effects, and identifies pulegone as a promising candidate active constituent. The pronounced nephroprotective effect of high-dose pulegone, its structural preservation of renal tissue, and its convergence with the essential oil’s own protective effects collectively support its further investigation as a candidate compound for renal cytoprotection in the context of aminoglycoside therapy.

## Data Availability

Data are available as Supplementary Materials.

## Supplementary Materials

The following supporting information can be downloaded at: https://www.mdpi.com/article/10.3390/antiox15101322/s1.

## Abbreviations

The following abbreviations are used in this manuscript:

BUN	Blood urea nitrogen
DPPH	2,2-diphenyl-1-picrylhydrazyl
EE	Ethanol extract loaded microemulsion formulation of *C. niveum*
EO	Essential oil of *C. niveum*
MDA	Malondialdehyde
SOD	Superoxide dismutase
