# Supplementary material for: Protective Effect of Cyclotrichium niveum on Gentamicin-Induced Nephrotoxicity in Rats
Source: Antioxidants (Basel). 2026 Oct 14;15(10):1322. doi: 10.3390/antiox15101322 (PMC13700843; doi:10.3390/antiox15101322)
Supplement: Supplementary file 1 [file antioxidants-15-01322-s001.zip › data/Tables.pdf]

Table S5: IC_{50} values of antioxidant compounds whose DPPH scav. act. were investigated. Values expressed are means±S.D. of four measurements.

**Antioxidant**	**IC_{50}** (µg/mL)
Ascorbic acid (positive control)	2.646±0.567
Ethanol extract loaded ME (EE)	48.174±1.003
Pulegone	120.003±1.519
Essential oil (EO)	14.889±0.989

Table S6: The distribution of histopathological grading between the groups (5 representative animals included in each treatment group).

**Grade**	**C**	**G**	**G + P 50 mg**	**G + P 100 mg**	**G + EE 200 mg**	**G + EE 400 mg**	**G+EO 200 mg**	**G+EO 400 mg**
0	5	0	0	3	0	0	3	4
1	0	0	3	2	3	3	1	1
2	0	0	2	0	2	2	1	0
3	0	0	0	0	0	0	0	0
4	0	1	0	0	0	0	0	0
5	0	4	0	0	0	0	0	0

Control Group (C), Gentamicin Group (G), Gentamicin + Pulegone 50 mg Group (G+P 50 mg), Gentamicin + Pulegone 100 mg Group (G+P 100 mg), Gentamicin + EE 200 mg Group (G+ EE 200 mg), Gentamicin + EE 400 mg Group (G+ EE 400 mg), Gentamicin + EO 200 mg group (G+EO 200 mg) and Gentamicin + EO 400 mg group (G+EO 400 mg).
