# Supplementary material for: Protective Effect of Cyclotrichium niveum on Gentamicin-Induced Nephrotoxicity in Rats
Source: Antioxidants (Basel). 2026 Oct 14;15(10):1322. doi: 10.3390/antiox15101322 (PMC13700843; doi:10.3390/antiox15101322)
Supplement: Supplementary file 1 [file antioxidants-15-01322-s001.zip › Figure_S1.pdf]

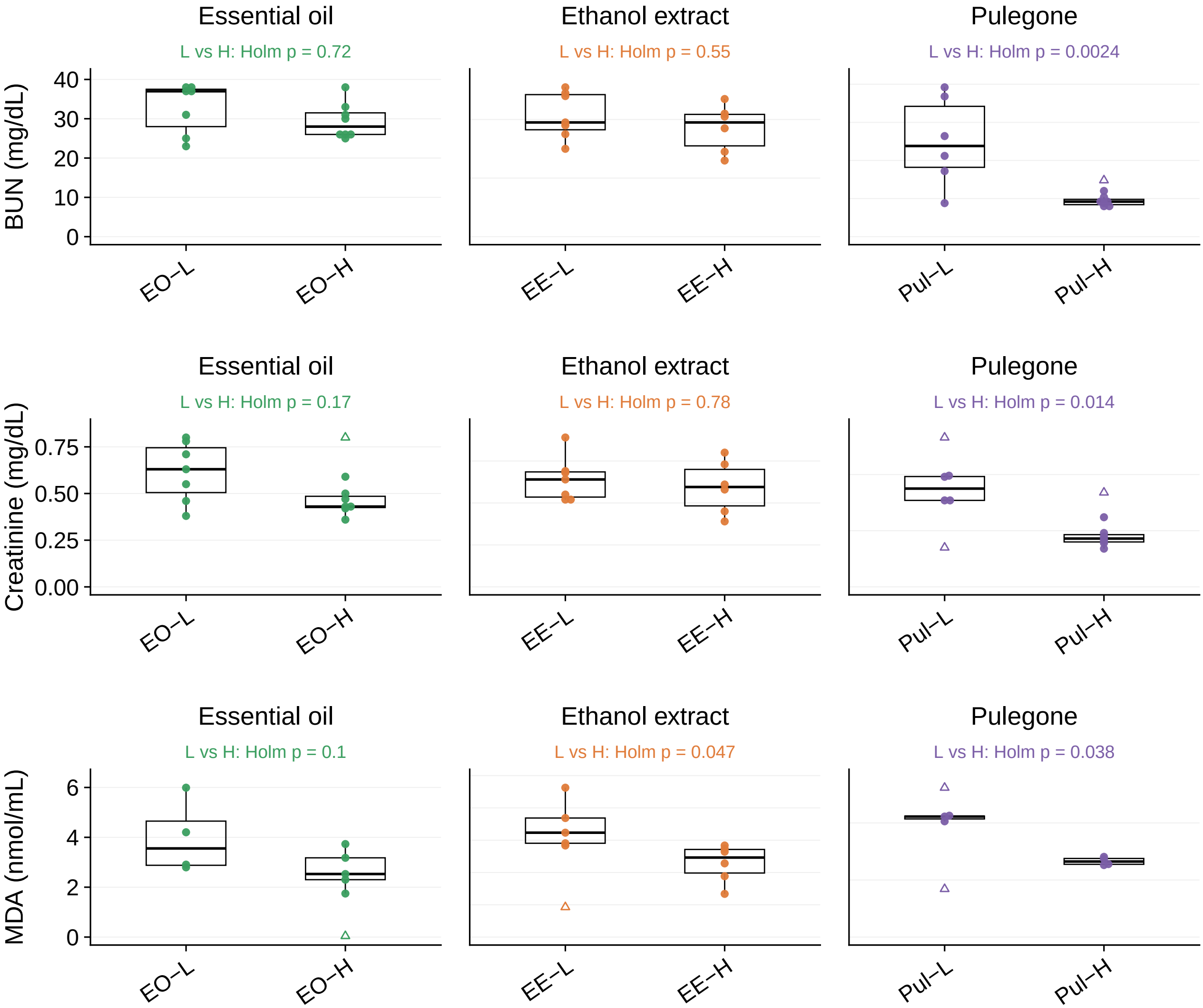
Essential oil
L vs H: Holm p = 0.72
Ethanol extract
L vs H: Holm p = 0.55
Pulegone
L vs H: Holm p = 0.0024
BUN (mg/dL)
40
30
20
10
0
EO–L
EO–H
EE–L
EE–H
Pul–L
Pul–H
Essential oil
L vs H: Holm p = 0.17
Ethanol extract
L vs H: Holm p = 0.78
Pulegone
L vs H: Holm p = 0.014
Creatinine (mg/dL)
0.75
0.50
0.25
0.00
EO–L
EO–H
EE–L
EE–H
Pul–L
Pul–H
Essential oil
L vs H: Holm p = 0.1
Ethanol extract
L vs H: Holm p = 0.047
Pulegone
L vs H: Holm p = 0.038
MDA (nmol/mL)
6
4
2
0
EO–L
EO–H
EE–L
EE–H
Pul–L
Pul–H
