# Supplementary material for: Protective Effect of Cyclotrichium niveum on Gentamicin-Induced Nephrotoxicity in Rats
Source: Antioxidants (Basel). 2026 Oct 14;15(10):1322. doi: 10.3390/antiox15101322 (PMC13700843; doi:10.3390/antiox15101322)
Supplement: Supplementary file 1 [file antioxidants-15-01322-s001.zip › Figure_S2.pdf]

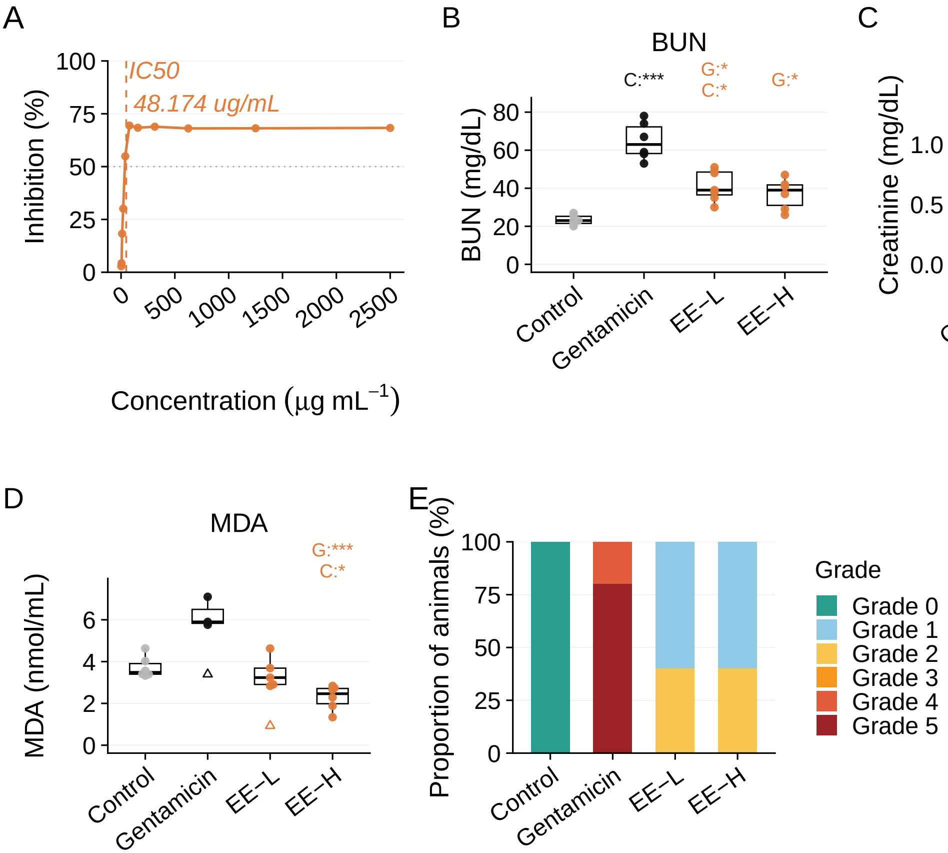
A
IC50
48.174 ug/mL
Inhibition (%)
Concentration (μg mL⁻¹)
B
BUN
BUN (mg/dL)
C:***
G:*
C:*
G:*
C
Creatinine
Creatinine (mg/dL)
C:***
C:**
C:*
D
MDA
MDA (nmol/mL)
G:***
C:*
E
Proportion of animals (%)
Control
Gentamicin
EE–L
EE–H
Grade
Grade 0
Grade 1
Grade 2
Grade 3
Grade 4
Grade 5
