# Supplementary material for: Protective Effect of Cyclotrichium niveum on Gentamicin-Induced Nephrotoxicity in Rats
Source: Antioxidants (Basel). 2026 Oct 14;15(10):1322. doi: 10.3390/antiox15101322 (PMC13700843; doi:10.3390/antiox15101322)
Supplement: Supplementary file 1 [file antioxidants-15-01322-s001.zip › Supplementary_Note_S1.pdf]

```
Analysis log — 2026-09-22 23:59:10
Main analysis: six groups (EE de-scoped to supplement)
Primary IQR approach: flagged IQR outliers REMOVED
Correction: Holm within marker x family
Primary inference: Kruskal-Wallis + Dunn contrasts
Supportive Welch estimates: ON

=== IQR OUTLIER FLAGS (k = 1.5 ) ===
   Measure      Group  Value
1      BUN     G+P100 37.000
2  CREATIN    G+UY400  0.800
3  CREATIN      G+P50  0.350
4  CREATIN      G+P50  1.330
5  CREATIN     G+P100  0.840
6      MDA Gentamicin  3.393
7      MDA    G+Et200  0.927
8      MDA    G+UY400  0.041
9      MDA      G+P50  1.684
10     MDA      G+P50  5.233
(written to outliers_excluded.csv)


############ PRIMARY ANALYSIS ############
IQR outliers REMOVED from primary statistics.

====== BUN (6 groups) ======
       Group  med    q1    q3 n
1    Control 23.0 21.50 25.25 6
2 Gentamicin 63.0 58.25 72.25 6
3       EO-L 37.0 28.00 37.50 7
4       EO-H 28.0 26.00 31.50 8
5      Pul-L 59.5 45.50 85.50 6
6      Pul-H 23.0 21.00 24.50 7
Kruskal-Wallis: H(5)=25.081, p=0.00013, epsilon^2=0.643

====== CREATIN (6 groups) ======
       Group   med     q1     q3 n
1    Control 0.300 0.2725 0.3425 6
2 Gentamicin 0.710 0.6400 0.8925 6
3       EO-L 0.630 0.5050 0.7450 7
4       EO-H 0.430 0.4250 0.4850 7
5      Pul-L 0.875 0.7700 0.9825 4
6      Pul-H 0.430 0.4000 0.4650 7
Kruskal-Wallis: H(5)=26.985, p=0.00006, epsilon^2=0.750

====== MDA (6 groups) ======
       Group    med      q1      q3 n
1    Control 3.4820 3.39750 3.90550 6
2 Gentamicin 5.8980 5.83050 6.49850 3
3       EO-L 3.5540 2.87775 4.64975 4
4       EO-H 2.5280 2.30100 3.17800 5
5      Pul-L 4.2220 4.13850 4.23700 3
6      Pul-H 2.6465 2.54850 2.75450 4
Kruskal-Wallis: H(5)=16.282, p=0.00608, epsilon^2=0.678
```

```
There were 14 warnings (use warnings() to see them)

#### Comparisons (PRIMARY INFERENCE: Dunn, Holm-adjusted) ####
   Measure     Family  Reference Treatment_label Fold_change
Cliff_delta        p_raw       p_holm sig
1      BUN  induction    Control      Gentamicin       2.739
1.000 1.947111e-04 1.947111e-04 ***
2      BUN   efficacy Gentamicin            EO-L       0.587
-1.000 3.967871e-02 7.935742e-02  ns
3      BUN vs_control    Control            EO-L       1.609
0.762 7.039697e-02 2.111909e-01  ns
4      BUN   efficacy Gentamicin            EO-H       0.444
-1.000 1.397269e-02 4.191806e-02   *
5      BUN vs_control    Control            EO-H       1.217
0.729 1.272434e-01 2.544868e-01  ns
6      BUN   efficacy Gentamicin           Pul-L       0.944
-0.139 4.804701e-01 4.804701e-01  ns
7      BUN vs_control    Control           Pul-L       2.587
0.778 2.525789e-03 1.010315e-02   *
8      BUN   efficacy Gentamicin           Pul-H       0.365
-1.000 7.101530e-05 2.840612e-04 ***
9      BUN vs_control    Control           Pul-H       1.000
-0.071 9.152394e-01 9.152394e-01  ns
10     BUN       dose    G+UY200            EO-H       0.757
-0.250 7.236463e-01 7.236463e-01  ns
11     BUN       dose      G+P50           Pul-H       0.387
-0.786 1.192458e-03 2.384916e-03  **
12 CREATIN  induction    Control      Gentamicin       2.367
1.000 3.974096e-05 3.974096e-05 ***
13 CREATIN   efficacy Gentamicin            EO-L       0.887
-0.452 3.121864e-01 6.243729e-01  ns
14 CREATIN vs_control    Control            EO-L       2.100
1.000 1.140145e-03 3.420435e-03  **
15 CREATIN   efficacy Gentamicin            EO-H       0.606
-0.952 1.929861e-02 5.789584e-02  ns
16 CREATIN vs_control    Control            EO-H       1.433
0.976 5.430715e-02 1.086143e-01  ns
17 CREATIN   efficacy Gentamicin           Pul-L       1.232
0.333 6.762143e-01 6.762143e-01  ns
18 CREATIN vs_control    Control           Pul-L       2.917
1.000 4.261614e-05 1.704646e-04 ***
19 CREATIN   efficacy Gentamicin           Pul-H       0.606
-0.952 1.031226e-02 4.124906e-02   *
20 CREATIN vs_control    Control           Pul-H       1.433
0.905 8.933580e-02 1.086143e-01  ns
21 CREATIN       dose    G+UY200            EO-H       0.683
-0.592 1.665616e-01 1.665616e-01  ns
22 CREATIN       dose      G+P50           Pul-H       0.491
-1.000 6.788981e-03 1.357796e-02   *
23     MDA  induction    Control      Gentamicin       1.694
1.000 8.962892e-02 8.962892e-02  ns
24     MDA   efficacy Gentamicin            EO-L       0.603
-0.667 1.160785e-01 2.321570e-01  ns
25     MDA vs_control    Control            EO-L       1.021
```

```
-0.083 1.000000e+00 1.000000e+00  ns
26     MDA   efficacy Gentamicin            EO-H       0.429
-1.000 1.630012e-03 6.044322e-03  **
27     MDA vs_control    Control            EO-H       0.726
-0.733 6.913494e-02 2.326637e-01  ns
28     MDA   efficacy Gentamicin           Pul-L       0.716
-1.000 4.374064e-01 4.374064e-01  ns
29     MDA vs_control    Control           Pul-L       1.213
0.667 4.233396e-01 8.466793e-01  ns
30     MDA   efficacy Gentamicin           Pul-H       0.449
-1.000 1.511081e-03 6.044322e-03  **
31     MDA vs_control    Control           Pul-H       0.760
-1.000 5.816593e-02 2.326637e-01  ns
32     MDA       dose    G+UY200            EO-H       0.711
-0.600 1.008726e-01 1.008726e-01  ns
33     MDA       dose      G+P50           Pul-H       0.627
-1.000 1.916285e-02 3.832569e-02   *

#### SUPPORTIVE WELCH ESTIMATION (does not drive figure stars) ####
   Measure     Family  Reference Treatment_label Mean_reference
Mean_treatment Welch_mean_diff Welch_CI_low Welch_CI_high
welch_p_raw welch_p_holm welch_sig
1      BUN  induction    Control      Gentamicin     23.3333333
64.8333333      41.5000000  31.21117066  51.788829341 7.516246e-05
7.516246e-05       ***
2      BUN   efficacy Gentamicin            EO-L     64.8333333
32.7142857     -32.1190476 -42.85093451 -21.387160723 1.039246e-04
3.117737e-04       ***
3      BUN vs_control    Control            EO-L     23.3333333
32.7142857       9.3809524   3.24392489  15.517979871 7.568045e-03
3.027218e-02         *
4      BUN   efficacy Gentamicin            EO-H     64.8333333
29.3750000     -35.4583333 -45.79950398 -25.117162684 1.063327e-04
3.117737e-04       ***
5      BUN vs_control    Control            EO-H     23.3333333
29.3750000       6.0416667   1.77085779  10.312475548 9.632623e-03
3.027218e-02         *
6      BUN   efficacy Gentamicin           Pul-L     64.8333333
62.3333333      -2.5000000 -33.09834266  28.098342657 8.487317e-01
8.487317e-01        ns
7      BUN vs_control    Control           Pul-L     23.3333333
62.3333333      39.0000000   8.41674989  69.583250107 2.185831e-02
4.371662e-02         *
8      BUN   efficacy Gentamicin           Pul-H     64.8333333
23.4285714     -41.4047619 -51.70788983 -31.101633978 5.805142e-05
2.322057e-04       ***
9      BUN vs_control    Control           Pul-H     23.3333333
23.4285714       0.0952381  -3.75086153   3.941337724 9.574768e-01
9.574768e-01        ns
10     BUN       dose    G+UY200            EO-H     32.7142857
29.3750000      -3.3392857  -9.78797251   3.109401081 2.774441e-01
2.774441e-01        ns
11     BUN       dose      G+P50           Pul-H     62.3333333
23.4285714     -38.9047619 -69.47822983  -8.331293982 2.196538e-02
```

```
4.393076e-02         *
12 CREATIN  induction    Control      Gentamicin      0.3050000
0.8000000       0.4950000   0.23217963   0.757820372 4.319825e-03
4.319825e-03        **
13 CREATIN   efficacy Gentamicin            EO-L      0.8000000
0.6157143      -0.1842857  -0.45726879   0.088697363 1.589862e-01
3.179724e-01        ns
14 CREATIN vs_control    Control            EO-L      0.3050000
0.6157143       0.3107143   0.16135511   0.460073461 1.679714e-03
4.690996e-03        **
15 CREATIN   efficacy Gentamicin            EO-H      0.8000000
0.4571429      -0.3428571  -0.60556029  -0.080153995 1.911136e-02
6.565808e-02        ns
16 CREATIN vs_control    Control            EO-H      0.3050000
0.4571429       0.1521429   0.07840200   0.225883715 9.649844e-04
3.859938e-03        **
17 CREATIN   efficacy Gentamicin           Pul-L      0.8000000
0.8775000       0.0775000  -0.20113970   0.356139702 5.367142e-01
5.367142e-01        ns
18 CREATIN vs_control    Control           Pul-L      0.3050000
0.8775000       0.5725000   0.38291337   0.762086626 1.563665e-03
4.690996e-03        **
19 CREATIN   efficacy Gentamicin           Pul-H      0.8000000
0.4457143      -0.3542857  -0.61737098  -0.091200444 1.641452e-02
6.565808e-02        ns
20 CREATIN vs_control    Control           Pul-H      0.3050000
0.4457143       0.1407143   0.05431125   0.227117322 4.973093e-03
4.973093e-03        **
21 CREATIN       dose    G+UY200            EO-H      0.6157143
0.4571429      -0.1585714  -0.31086232  -0.006280535 4.302790e-02
4.302790e-02         *
22 CREATIN       dose      G+P50           Pul-H      0.8775000
0.4457143      -0.4317857  -0.61549077  -0.248080659 1.946833e-03
3.893667e-03        **
23     MDA  induction    Control      Gentamicin      3.7233333
6.2533333       2.5300000   1.02700113   4.032998872 1.269891e-02
1.269891e-02         *
24     MDA   efficacy Gentamicin            EO-L      6.2533333
3.9735000      -2.2798333  -4.54999065  -0.009676021 4.936418e-02
7.396112e-02        ns
25     MDA vs_control    Control            EO-L      3.7233333
3.9735000       0.2501667  -2.02968826   2.530021590 7.646847e-01
7.646847e-01        ns
26     MDA   efficacy Gentamicin            EO-H      6.2533333
2.6966000      -3.5567333  -5.00885626  -2.104610406 1.856801e-03
7.427204e-03        **
27     MDA vs_control    Control            EO-H      3.7233333
2.6966000      -1.0267333  -1.98869186  -0.064774805 3.969983e-02
1.190995e-01        ns
28     MDA   efficacy Gentamicin           Pul-L      6.2533333
4.1763333      -2.0770000  -3.85400508  -0.299994923 3.698056e-02
7.396112e-02        ns
29     MDA vs_control    Control           Pul-L      3.7233333
4.1763333       0.4530000  -0.08240575   0.988405749 8.345014e-02
```

```
1.669003e-01        ns
30     MDA   efficacy Gentamicin           Pul-H      6.2533333
2.6565000      -3.5968333  -5.36084039  -1.832826278 1.196237e-02
3.588712e-02         *
31     MDA vs_control    Control           Pul-H      3.7233333
2.6565000      -1.0668333  -1.60254346  -0.531123210 2.752875e-03
1.101150e-02         *
32     MDA       dose    G+UY200            EO-H      3.9735000
2.6966000      -1.2769000  -3.49691391   0.943113910 1.899913e-01
1.899913e-01        ns
33     MDA       dose      G+P50           Pul-H      4.1763333
2.6565000      -1.5198333  -1.75805913  -1.281607539 1.602309e-05
3.204619e-05       ***

########## SENSITIVITY ANALYSIS ##########
All non-QC-failed observations RETAINED.
Warning messages:
1: In cliff.delta.default(treat, ref) :
  The samples are fully disjoint, using approximate Confidence
Interval estimation
2: In cliff.delta.default(treat, ref) :
  The samples are fully disjoint, using approximate Confidence
Interval estimation
3: In cliff.delta.default(treat, ref) :
  The samples are fully disjoint, using approximate Confidence
Interval estimation
4: In cliff.delta.default(treat, ref) :
  The samples are fully disjoint, using approximate Confidence
Interval estimation
5: In cliff.delta.default(treat, ref) :
  The samples are fully disjoint, using approximate Confidence
Interval estimation
6: In cliff.delta.default(treat, ref) :
  The samples are fully disjoint, using approximate Confidence
Interval estimation
7: In cliff.delta.default(treat, ref) :
  The samples are fully disjoint, using approximate Confidence
Interval estimation
8: In cliff.delta.default(treat, ref) :
  The samples are fully disjoint, using approximate Confidence
Interval estimation

fold_change_summary.csv written.
welch_supportive_primary.csv written (supportive only; not used for
figure stars).
sensitivity_analysis.csv written.

Comparisons whose significance label changes under the alternate IQR
approach:
  Measure     Family  Reference Treatment_label Fold_primary
pHolm_primary sig_primary Welch_diff_primary Welch_CI_low_primary
Welch_CI_high_primary Welch_pHolm_primary
1     BUN vs_control    Control           Pul-L        2.587
0.010103154          *        39.0000000          8.41674989
```

```
69.58325011        4.371662e-02
2 CREATIN vs_control    Control            EO-H       1.433
0.108614302          ns         0.1521429          0.07840200
0.22588371        3.859938e-03
3 CREATIN   efficacy Gentamicin           Pul-H       0.606
0.041249059           *        -0.3542857         -0.61737098
-0.09120044        6.565808e-02
4 CREATIN vs_control    Control           Pul-H       1.433
0.108614302          ns         0.1407143          0.05431125
0.22711732        4.973093e-03
5 CREATIN       dose      G+P50           Pul-H       0.491
0.013577962           *        -0.4317857         -0.61549077
-0.24808066        3.893667e-03
6     MDA   efficacy Gentamicin           Pul-H       0.449
0.006044322          **        -3.5968333         -5.36084039
-1.83282628        3.588712e-02
7     MDA       dose      G+P50           Pul-H       0.627
0.038325692           *        -1.5198333         -1.75805913
-1.28160754        3.204619e-05
  Welch_sig_primary Fold_sens  pHolm_sens sig_sens Welch_diff_sens
Welch_CI_low_sens Welch_CI_high_sens Welch_pHolm_sens Welch_sig_sens
verdict Welch_verdict
1                 *     2.587 0.008617288       **        39.00000
8.41674989        69.58325011       0.04371662             *
CHANGES     unchanged
2                **     1.500 0.048247013        *         0.19500
0.07618499         0.31381501       0.01467179             *
CHANGES       CHANGES
3                ns     0.620 0.140484010       ns        -0.30500
-0.57537760        -0.03462240       0.12612464            ns
CHANGES     unchanged
4                **     1.467 0.048247013        *         0.19000
0.05251242         0.32748758       0.01595524             *
CHANGES       CHANGES
5                **     0.503 0.079678054       ns        -0.37000
-0.71312903        -0.02687097       0.07635208            ns
CHANGES       CHANGES
6                 *     0.454 0.018403688        *        -2.88175
-5.33788810        -0.42561190       0.10004863            ns
CHANGES       CHANGES
7               ***     0.627 0.112699698       ns        -1.23270
-2.86202174         0.39662174       0.20866121            ns
CHANGES       CHANGES
Histopathology grades read from data/histopathology.csv

=== HISTOPATHOLOGY GRADE DISTRIBUTION ===
       Group G0 G1 G2 G3 G4 G5 n
1    Control  5  0  0  0  0  0 5
2 Gentamicin  0  0  0  0  1  4 5
3    G+UY200  3  1  1  0  0  0 5
4    G+UY400  4  1  0  0  0  0 5
5    G+Et200  0  3  2  0  0  0 5
6    G+Et400  0  3  2  0  0  0 5
7      G+P50  0  3  2  0  0  0 5
```

```
8     G+P100  3  2  0  0  0  0 5
WARNING: identical histopathology grade vectors detected:
  G+Et200 == G+Et400
  G+Et200 == G+P50
  G+Et400 == G+P50

=== HISTOPATHOLOGY (main six groups): H(5)=21.374, p=0.00069,
epsilon^2=0.737 ===
       family         g1         g2 Cliff_delta        p_raw
p_holm sig
1   induction    Control Gentamicin        1.00 0.0001071433
0.0001071433 ***
2    efficacy Gentamicin       EO-L       -1.00 0.0046856289
0.0093712578  **
3  vs_control    Control       EO-L        0.40 0.2955929164
0.8867787493  ns
4    efficacy Gentamicin       EO-H       -1.00 0.0005653852
0.0022615408  **
5  vs_control    Control       EO-H        0.20 0.6700203971
0.8867787493  ns
6    efficacy Gentamicin      Pul-L       -1.00 0.1751512242
0.1751512242  ns
7  vs_control    Control      Pul-L        1.00 0.0118029386
0.0472117542   *
8    efficacy Gentamicin      Pul-H       -1.00 0.0025146061
0.0075438183  **
9  vs_control    Control      Pul-H        0.40 0.3940808841
0.8867787493  ns
10       dose       EO-L       EO-H       -0.24 0.5353822720
0.5353822720  ns
11       dose      Pul-L      Pul-H       -0.76 0.0957648379
0.1915296758  ns
Warning messages:
1: In cliff.delta.default(treat, ref) :
  The samples are fully disjoint, using approximate Confidence
Interval estimation
2: In cliff.delta.default(treat, ref) :
  The samples are fully disjoint, using approximate Confidence
Interval estimation
3: In cliff.delta.default(treat, ref) :
  The samples are fully disjoint, using approximate Confidence
Interval estimation
4: In cliff.delta.default(treat, ref) :
  The samples are fully disjoint, using approximate Confidence
Interval estimation
5: In cliff.delta.default(treat, ref) :
  The samples are fully disjoint, using approximate Confidence
Interval estimation
6: In cliff.delta.default(treat, ref) :
  The samples are fully disjoint, using approximate Confidence
Interval estimation

############ EE SUPPLEMENTARY ANALYSIS (4 groups) ############
EE primary uses the SAME IQR approach as the main biochemical
```

analysis.

```
====== BUN (4 groups) ======
       Group med    q1    q3 n
1    Control  23 21.50 25.25 6
2 Gentamicin  63 58.25 72.25 6
3       EE-L  39 36.50 48.50 7
4       EE-H  39 31.00 41.75 6
Kruskal-Wallis: H(3)=19.841, p=0.00018, epsilon^2=0.827

====== CREATIN (4 groups) ======
       Group   med     q1     q3 n
1    Control 0.300 0.2725 0.3425 6
2 Gentamicin 0.710 0.6400 0.8925 6
3       EE-L 0.640 0.5350 0.6850 7
4       EE-H 0.595 0.4825 0.7000 6
Kruskal-Wallis: H(3)=14.717, p=0.00208, epsilon^2=0.613

====== MDA (4 groups) ======
       Group    med     q1     q3 n
1    Control 3.4820 3.3975 3.9055 6
2 Gentamicin 5.8980 5.8305 6.4985 3
3       EE-L 3.2330 2.9050 3.6870 5
4       EE-H 2.4625 1.9835 2.7135 6
Kruskal-Wallis: H(3)=15.483, p=0.00145, epsilon^2=0.815
There were 11 warnings (use warnings() to see them)
Warning messages:
1: In cliff.delta.default(treat, ref) :
  The samples are fully disjoint, using approximate Confidence
Interval estimation
2: In cliff.delta.default(treat, ref) :
  The samples are fully disjoint, using approximate Confidence
Interval estimation
3: In cliff.delta.default(treat, ref) :
  The samples are fully disjoint, using approximate Confidence
Interval estimation
4: In cliff.delta.default(treat, ref) :
  The samples are fully disjoint, using approximate Confidence
Interval estimation
5: In cliff.delta.default(treat, ref) :
  The samples are fully disjoint, using approximate Confidence
Interval estimation
6: In cliff.delta.default(treat, ref) :
  The samples are fully disjoint, using approximate Confidence
Interval estimation
7: In cliff.delta.default(treat, ref) :
  The samples are fully disjoint, using approximate Confidence
Interval estimation
8: In cliff.delta.default(treat, ref) :
  The samples are fully disjoint, using approximate Confidence
Interval estimation
9: In cliff.delta.default(treat, ref) :
  The samples are fully disjoint, using approximate Confidence
Interval estimation
```

```
=== HISTOPATHOLOGY (EE supplement): H(3)=17.032, p=0.00070,
epsilon^2=0.896 ===
      family         g1         g2 Cliff_delta        p_raw
p_holm sig
1  induction    Control Gentamicin           1 0.0000367576
0.0000367576 ***
2   efficacy Gentamicin       EE-L          -1 0.0390666225
0.0781332450  ns
3 vs_control    Control       EE-L           1 0.0390666225
0.0781332450  ns
4   efficacy Gentamicin       EE-H          -1 0.0390666225
0.0781332450  ns
5 vs_control    Control       EE-H           1 0.0390666225
0.0781332450  ns
6       dose       EE-L       EE-H           0 1.0000000000
1.0000000000  ns
Warning messages:
1: In cliff.delta.default(treat, ref) :
  The samples are fully disjoint, using approximate Confidence
Interval estimation
2: In cliff.delta.default(treat, ref) :
  The samples are fully disjoint, using approximate Confidence
Interval estimation
3: In cliff.delta.default(treat, ref) :
  The samples are fully disjoint, using approximate Confidence
Interval estimation
4: In cliff.delta.default(treat, ref) :
  The samples are fully disjoint, using approximate Confidence
Interval estimation
5: In cliff.delta.default(treat, ref) :
  The samples are fully disjoint, using approximate Confidence
Interval estimation
EE_supplementary_stats.csv written.
EE_sensitivity_analysis.csv written.
results/figures/Figure1 saved (174 x 130 mm)
Warning messages:
1: In grid.Call.graphics(C_text, as.graphicsAnnot(x$label), x$x,
x$y,  :
  conversion failure on 'α-Elemene' in 'mbcsToSbcs': for α (U+03B1)
2: In grid.Call.graphics(C_text, as.graphicsAnnot(x$label), x$x,
x$y,  :
  conversion failure on 'α-Myrcene' in 'mbcsToSbcs': for α (U+03B1)
3: In grid.Call.graphics(C_text, as.graphicsAnnot(x$label), x$x,
x$y,  :
  conversion failure on 'α-Pinene' in 'mbcsToSbcs': for α (U+03B1)
4: In grid.Call.graphics(C_text, as.graphicsAnnot(x$label), x$x,
x$y,  :
  conversion failure on '2-β-Pinene' in 'mbcsToSbcs': for β (U+03B2)
5: In grid.Call.graphics(C_text, as.graphicsAnnot(x$label), x$x,
x$y,  :
  conversion failure on '2.646 µg/mL' in 'mbcsToSbcs': for µ
(U+03BC)
6: In grid.Call.graphics(C_text, as.graphicsAnnot(x$label), x$x,
```

```
x$y,  :
  conversion failure on '14.889 µg/mL' in 'mbcsToSbcs': for µ
(U+03BC)
7: In grid.Call.graphics(C_text, as.graphicsAnnot(x$label), x$x,
x$y,  :
  conversion failure on '120.003 µg/mL' in 'mbcsToSbcs': for µ
(U+03BC)
8: In grid.Call.graphics(C_text, as.graphicsAnnot(x$label), x$x,
x$y,  :
  for '[insert structure —' in 'mbcsToSbcs': - substituted for —
(U+2014)
Warning messages:
1: In unit(c(t, r, b, l), unit) : NAs introduced by coercion
2: In unit(c(t, r, b, l), unit) : NAs introduced by coercion
3: In unit(c(t, r, b, l), unit) : NAs introduced by coercion
results/figures/Figure2 saved (174 x 78 mm)
Warning messages:
1: In unit(c(t, r, b, l), unit) : NAs introduced by coercion
2: In unit(c(t, r, b, l), unit) : NAs introduced by coercion
3: In unit(c(t, r, b, l), unit) : NAs introduced by coercion
4: In unit(c(t, r, b, l), unit) : NAs introduced by coercion
5: In unit(c(t, r, b, l), unit) : NAs introduced by coercion
6: In unit(c(t, r, b, l), unit) : NAs introduced by coercion
7: In unit(c(t, r, b, l), unit) : NAs introduced by coercion
8: In unit(c(t, r, b, l), unit) : NAs introduced by coercion
9: In unit(c(t, r, b, l), unit) : NAs introduced by coercion
results/figures/FigureS_dose saved (174 x 150 mm)
results/figures/Figure3 saved (135 x 90 mm)
Warning messages:
1: In unit(c(t, r, b, l), unit) : NAs introduced by coercion
2: In unit(c(t, r, b, l), unit) : NAs introduced by coercion
3: In unit(c(t, r, b, l), unit) : NAs introduced by coercion
results/figures/FigureS_EE saved (174 x 125 mm)

=== ANALYSIS INTERPRETATION NOTE ===
Figure stars are based ONLY on the prespecified Dunn/Holm primary
inference.
Welch results are supportive estimates/robustness checks and are
never combined with Dunn into a decision rule.
C: comparisons test difference from Control.
Histopathology is analyzed as ordinal data with rank-based tests
only.
```
