# Supplementary material for: Protective Effect of Cyclotrichium niveum on Gentamicin-Induced Nephrotoxicity in Rats
Source: Antioxidants (Basel). 2026 Oct 14;15(10):1322. doi: 10.3390/antiox15101322 (PMC13700843; doi:10.3390/antiox15101322)
Supplement: Supplementary file 1 [file antioxidants-15-01322-s001.zip › Supplementary_Note_S2.pdf]

Supplementary analysis on Ethanol Extract-Loaded Microemulsion of *C. niveum*

Methodology

Ethanol extraction. For ethanol extraction, 150 g of air-dried aerial parts of *C. niveum* was ground to a fine powder and macerated with 750 mL ethanol for 24 h at room temperature on a shaker. The extraction was performed in three batches; the resulting extracts were filtered sequentially through cheesecloth and Whatman No. 1 filter paper, concentrated under reduced pressure using a rotary evaporator (ISOLAB, Laborgeräte GmbH) at 40°C, and combined into a single pooled dry extract used throughout the study. The pooled extract was stored at -80°C until use. A microemulsion (ME) delivery system was developed to improve oral absorption and bioactivity of the extract. Pseudo-ternary phase diagrams were constructed by water titration of surfactant/co-surfactant (S/CoS) mixtures and oil phase at room temperature. Isopropyl myristate served as the oil phase and a Cremophor® RH 40 / propylene glycol mixture (2:1) as the S/CoS system. These were combined at weight ratios of 1:9, 2:8, 3:7, 4:6, 5:5, 6:4, 7:3, 8:2, and 9:1 (oil : S/CoS), and water was titrated dropwise until a transparent, single-phase microemulsion formed, indicating the microemulsion region of the phase diagram. Formulations were subsequently prepared at room temperature with mechanical stirring, and the ethanol extract was incorporated at 5% w/w into the microemulsion base. The resulting EE formulation contained 5% (w/w) dried ethanol extract. The EE formulation was characterized for visual clarity and centrifugation stability. Stability was assessed by centrifuging samples at 5000 rpm for 30 min at room temperature and visually inspecting for phase separation, and by subjecting samples to three heating-cooling cycles (4°C and 40°C, 48 h per cycle) followed by visual inspection for phase separation or creaming.

Experimental design. Eight rats were initially assigned to each experimental group. Some animals were lost during the course of the experiment, leaving 6-8 animals per group at the end of the study. All treatments were administered for eight days: (i) Control group (n = 6), receiving normal diet and drinking water; (ii) Gentamicin group (n = 6), receiving gentamicin at 100 mg/kg/day, intramuscularly (im); (iii) Gentamicin + EE 200 group (EE-L; n = 7), receiving gentamicin (100 mg/kg/day, im) co-administered with the EE formulation at 200 mg/kg/day, orally (gavage); (iv) Gentamicin + EE 400 group (EE-H; n = 6), receiving gentamicin (100 mg/kg/day, im) co-administered with the EE formulation at 400 mg/kg/day, orally (gavage). 24 hours after the final administration on day 8, animals were anesthetized with ketamine (Ketalar®, 40 mg/kg) and xylazine hydrochloride (Basilazin 2%, 5 mg/kg) and sacrificed. Intracardiac blood was collected for biochemical analysis, and the left kidney was removed via a midline abdominal incision for histopathological examination.

The rest followed the methodology from the main text.

Results

The ethanol extract provided additional, although less directly interpretable, evidence of the nephroprotective potential of *C. niveum*. Both EE doses reduced BUN, while the higher dose significantly reduced MDA, and histopathological examination showed substantially less severe renal injury than in the gentamicin group (Figure S2).

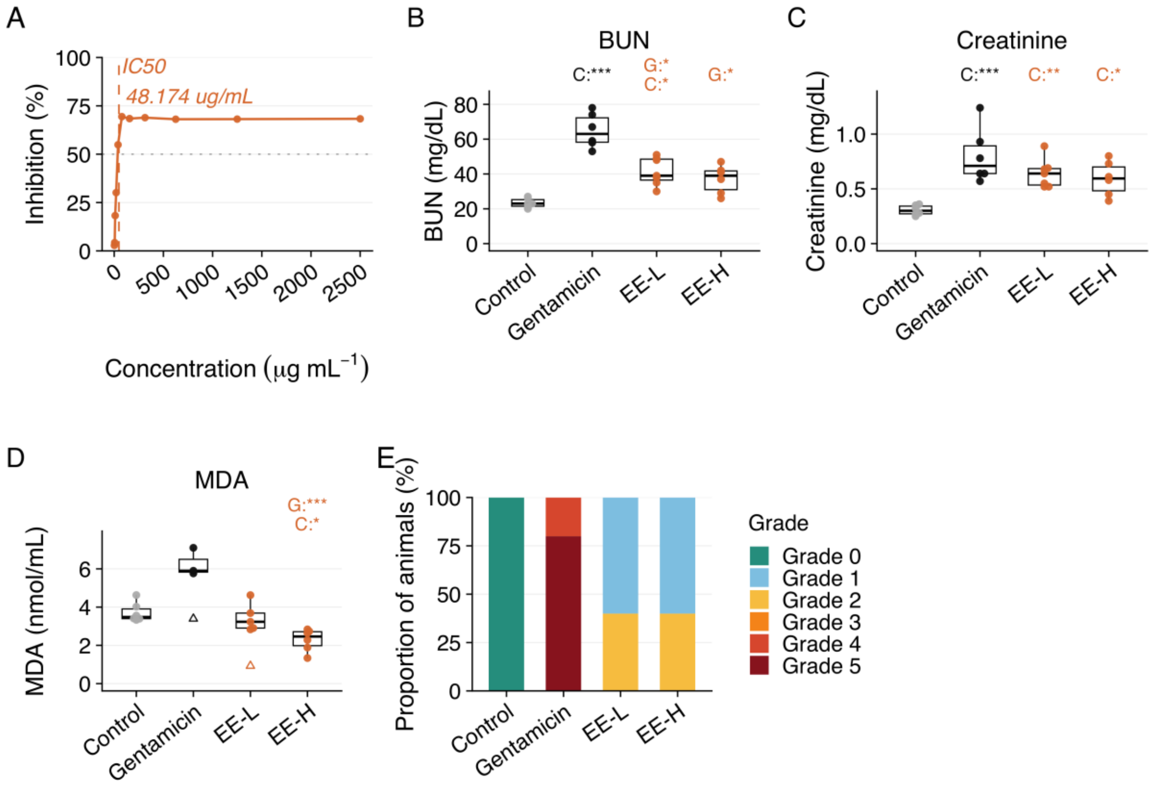

Figure S2. Antioxidant capacity of *C. niveum* ethanol extract and its effect on biochemical markers of gentamicin-induced nephrotoxicity. (A) DPPH radical scavenging activity (% inhibition) as a function of concentration (µg/mL). (B) Blood urea nitrogen (BUN, mg/dL), (C) serum creatinine (mg/dL), and (D) malondialdehyde (MDA, nmol/mL) across Control, Gentamicin, and treatment groups (EE-L/EE-H). Filled circles represent individual observations included in the primary statistical analysis, whereas open triangles indicate values flagged by the IQR criterion and excluded from the primary analysis but retained in the all-data sensitivity analysis. Boxes show the median and interquartile range. (E) Distribution of histopathological injury grades across the treatment groups, expressed as the proportion of animals per group (n=5 per group).

However, the chemical composition of the extract was not characterized, preventing attribution of these effects to pulegone or other specific constituents. In addition, because EE was administered in a microemulsion containing Cremophor RH40 and propylene glycol, potential effects of the vehicle on oxidative responses cannot be excluded in the absence of a corresponding blank microemulsion control. We therefore regard the EE findings as supportive evidence.
